# MicroRNA-146a promote cell migration and invasion in human colorectal cancer via carboxypeptidase M/src-FAK pathway

**DOI:** 10.18632/oncotarget.15158

**Published:** 2017-02-07

**Authors:** Di Lu, Qunyan Yao, Cheng Zhan, Zhang Le-Meng, Hongchun Liu, Yu Cai, Chuantao Tu, Xi Li, Yanting Zou, Shuncai Zhang

**Affiliations:** ^1^ Department of Gastroenterology, Zhongshan Hospital, Fudan University, Shanghai, 200032, China; ^2^ Department of Thoracic Surgery, Zhongshan Hospital, Fudan University, Shanghai, 200032, China; ^3^ Department of The Affiliated Cancer Hospital, Xiang Ya School of Medicine, Central South University, Changsha, Hunan, 410013, China

**Keywords:** miR-146a-5p, carboxypeptidase M, colorectal cancer, migration, invasion

## Abstract

Colorectal cancer (CRC) is one of the most common cancers worldwide, and microRNAs play important roles in CRC progression. This study aimed to investigate the roles of miR-146a-5p in human CRC and their molecular mechanisms. First, we found that miR-146a-5p was significantly upregulated in CRC tissues and promoted the migration of CRC cells. Then, we identified carboxypeptidase M (CPM) as a direct target of miR-146a-5p, and found that it inhibited the migration and invasion of CRC cells. Our results also showed that CPM expression was positively correlated with overall survival and negatively correlated with recurrence, lymph node invasion, and N stage. Furthermore, we demonstrated that both miR-146a-5p and CPM regulated Src and FAK expression, while the Src-FAK signaling pathway is widely known to be associated with the migration and invasion of multiple tumor cells. This study is the first to demonstrate the functional and mechanistic relationship of the miR-146a-5p/CPM/Src-FAK axis and its effect on the migration and invasion of CRC cells. Thus, miR-146a-5p represents potential targets for CRC diagnosis and therapy.

## INTRODUCTION

Colorectal cancer (CRC) is a major cause of cancer related deaths [1], and the third most commonly diagnosed cancer worldwide [2]. Despite recent advances in treatment, the prognosis of patients with colorectal cancer (CRC) remains substandard. Metastatic recurrence following curative surgery is the leading cause of mortality. Therefore, it is imperative to identify prognostic markers to predict the clinical outcome of CRC patients [3]. MicroRNAs (miRNAs) are a family of endogenous, small, noncoding RNA molecules mediating a wide range of biological processes, including differentiation, proliferation, and apoptosis [4]. miRNAs can function as either oncogenes or tumor suppressor genes, depending on the type of tumor or the cellular context [5], and their deregulation is frequent in human colorectal cancers (CRCs) and their deregulation is linked to cancer progression and clinical outcome [6, 7].

In this study, we found that miR-146a-5p was highly expressed in CRC, promoted cell migration and invasion of CRC cells. Then we further explored its molecular mechanisms and identified CPM (carboxypeptidase M) as a direct target of miR-146a-5p. Our results also showed that both miR-146a-5p and CPM could regulate the expression of Src and FAK. Thus, we hypothesize that miR-146a-5p downregulates the expression of CPM and activates the Src-FAK pathway to facilitate CRC cell migration and invasion. To our knowledge, this study is the first to demonstrate the functional and mechanistic relationship of the miR-146a-5p/CPM/Src-FAK axis and its effect on the migration and invasion of CRC cells.

## RESULTS

### miR-146a-5p was upregulated in CRC and promoted CRC migration

**Figure 1:**
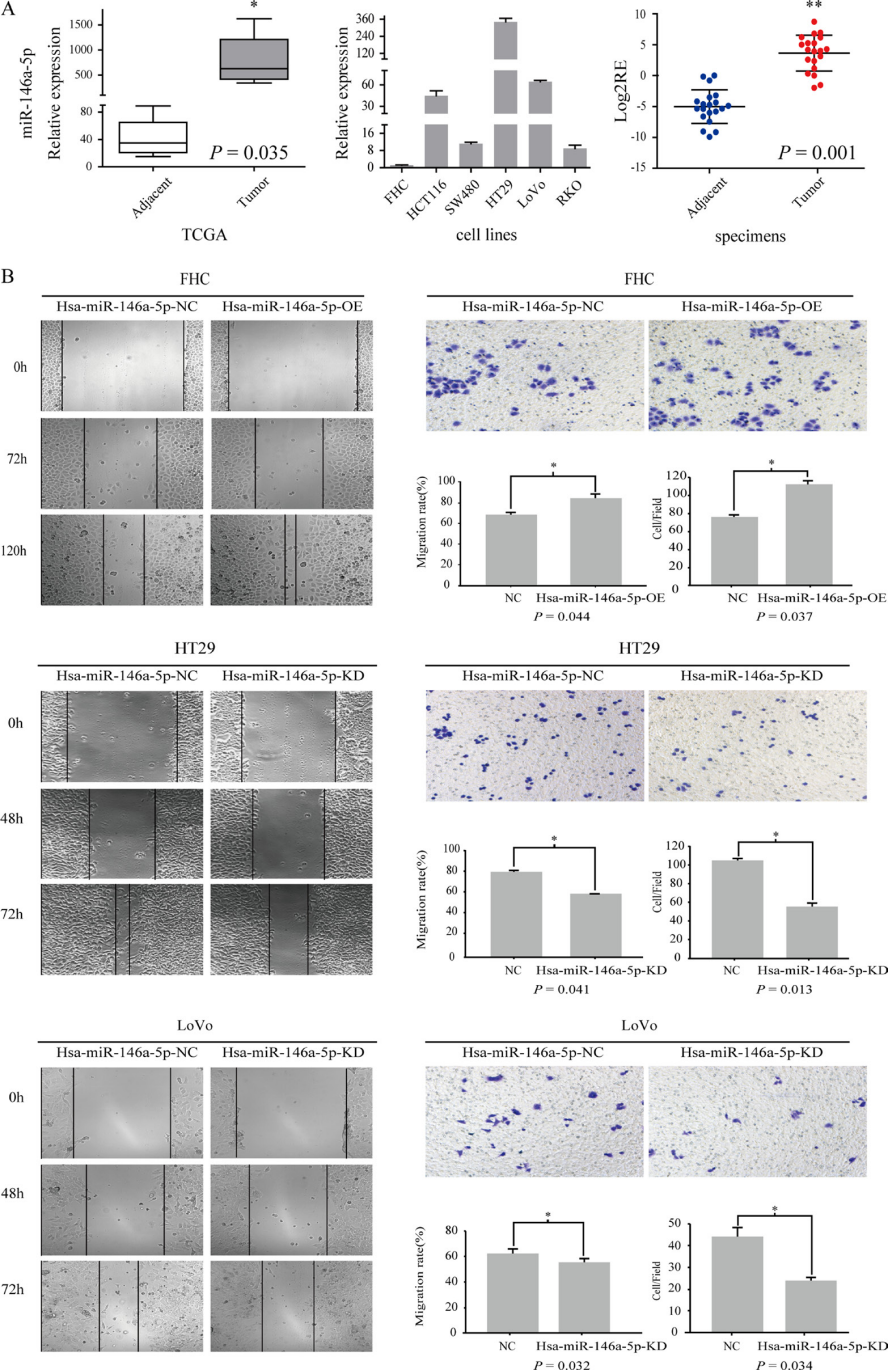
miR-146a-5p was upregulated in CRC and promoted CRC migration (**A**) The relative expression of miR-146a-5p in adjacent tissues and tumor tissues in the TCGA database. miR-146a-5p expression was detected in five CRC cell lines (HT29, SW480, LoVo, RKO, and HCT116) and an immobilized colonic epithelial cell line (FHC) (RT-PCR). The Log2RE (relative expression) of miR-146a-5p was detected in 20 pairs of specimens from CRC patients (RT-PCR). (**B**) The results of the scratch wound cell migration assay in FHC-miR-146a-5p NC (negative control)/miR-146a-5p OE (overexpression mimics), HT29-miR-146a-5p NC/miR-146a-5p KD (Knockdown, inhibitor), and LoVo-miR-146a-5p NC/miR-146a-5p KD cells. The results of the transwell migration assay for FHC-miR-146a-5p NC/OE, HT29-miR-146a-5p NC/KD, and LoVo-miR-146a-5p NC/KD cells. The statistical results of the scratch wound cell migration assay and transwell migration assay.

To determine the candidate miRNA biomarkers in CRC, we performed an integrative analysis of miRNA expression profiling comparing CRC tissues with paired adjacent tissues using the TCGA and UCSC cancer databases. We found that miR-146a-5p expression was significantly upregulated in CRC relative to that in adjacent tissues (*P* = 0.035) (Figure 1A). We tested miR-146a-5p expression in five CRC cell lines (HT29, HCT116, RKO, SW480, and LoNo) and one normal colon epithelial cell line (FHC) using RT-PCR. miR-146a-5p was upregulated in all CRC cell lines but not in FHC cells (Figure 1A). miR-146a-5p expression was upregulated (*P* = 0.001) in CRC tissues relative to that in adjacent tissues in 55% of the 20 patients (Figure 1A).

We overexpressed miR-146a-5p in FHC cells (OE) given its low expression in FHC and knocked it out in HT29 (KD) and LoVo cells (KD) given its relatively high expression in HT29 and LoVo cells. We found that the migration of miR-146a-5p-overexpressing FHC cells was enhanced compared with that in the control group (NC) (Figure 1B) (*P* < 0.05), whereas the opposite phenomenon occurred in miR-146a-5p-knockdown HT29 and LoVo cells (Figure 1B) (*P* < 0.05)

Moderate changes were noted in the colony forming abilities of FHC miR-146a-5p OE, HT29 miR-146a-5p KD and LoVo miR-146a-5p KD cells relative to the colony-forming abilities observed in their respective control groups (Supplementary Figure 1B).

### CPM was the target gene of miR-146a-5p

**Figure 2:**
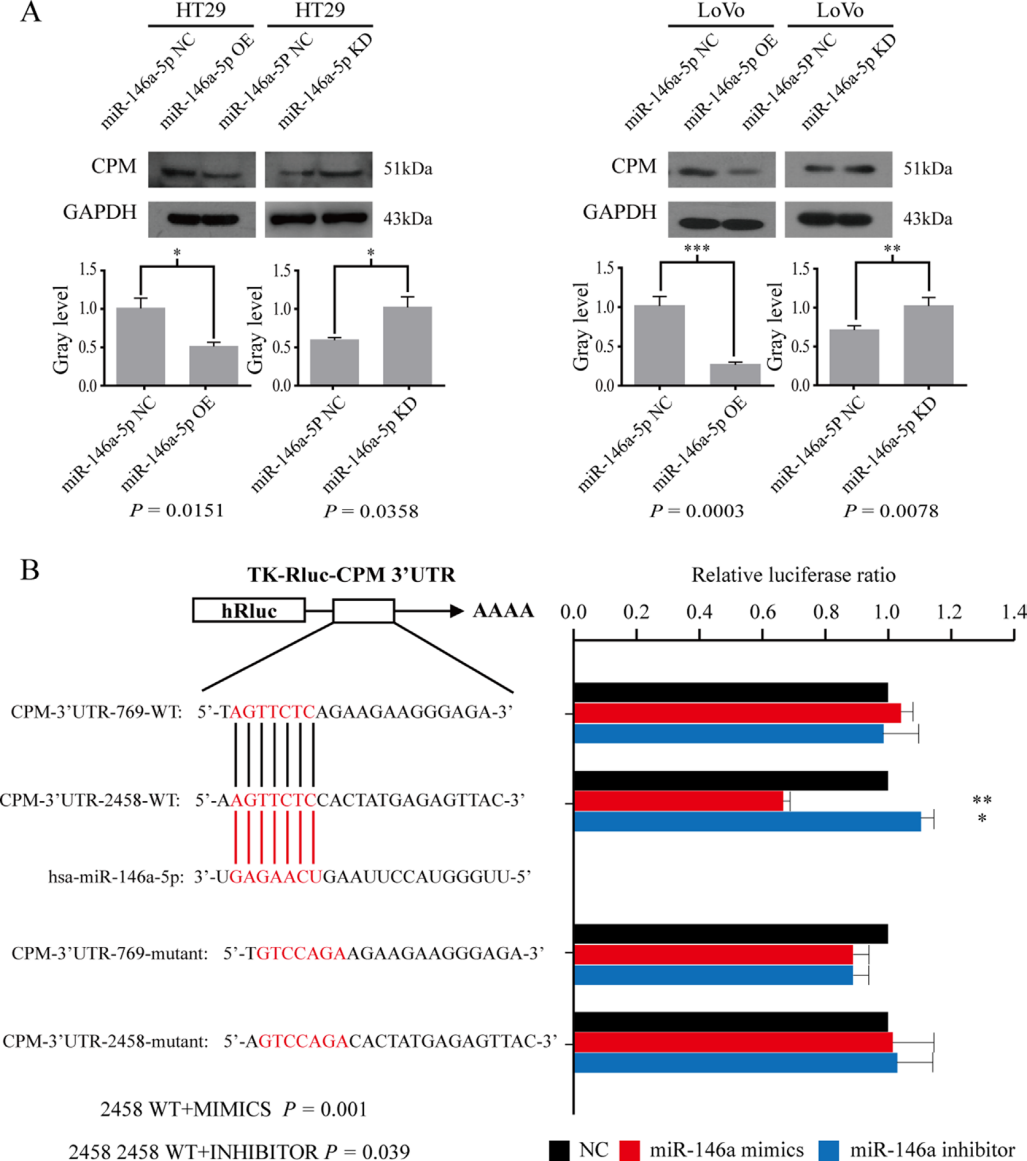
CPM is a target gene of miR-146a-5p (**A**) Western blot results for CPM in HT29-miR-146a-5p NC/OE/KD cells and the corresponding gray level, LoVo-miR-146a-5p NC/OE/KD and the corresponding gray level. (**B**) Luciferase assay results showing miR-146a-5p binding to CPM 3′UTR 2458 in LoVo cells (Welch's unpaired *t* test).

We selected CPM, CDC42BPA, KLF6, and CHD9 as the candidate targets of miR-146a-5p. qRT-PCR results in cell lines showed that CPM regulated by miR-146a-5p was the most significant in these four genes after the expression of miR-146a-5p was altered (Supplementary File). Therefore, our following experiments focus on CPM as a target for miR-146a. Western blot results further indicated that the overexpression of miR-146a-5p reduced CPM, and the inhibition of miR-146a-5p promoted the expression of CPM in HT29 and LoVo cells (Figure 2A). According to the bioinformatics analyses, we revealed that in CPM 3′ untranslated region (UTR) there were two binding sites for miR-146a-5p (CPM 3′UTR 2458 and CPM 3′UTR 769). The luciferase reporter assay results suggested that miR-146a-5p binds to CPM 3′UTR 2458 in LoVo cells (Figure 2B). Statistical results are presented in Table 1.

**Table 1:** The statistical results of luciferase assay

	mean	S.D	*p*
2458 WT	1	0	
2458 WT + MIMICS	0.668811	0.019692	0.001176
2458 WT + INHIBITOR	1.109035	0.038747	0.03961
2458 MUT	1	0	
2458 MUT + MIMICS	1.021552	0.131278	0.80288
2458 MUT + INHIBITOR	1.03028	0.116655	0.697037
769 WT	1	0	
769 WT + MIMICS	1.045496	0.045439	0.225017
769 WT + INHIBITOR	0.983758	0.131848	0.850814
769-MUT	1	0	
769 MUT + MIMICS	0.88658	0.055205	0.070696
769 MUT + INHIBITOR	0.893002	0.05451	0.076694

Each test was repeated three times.

Statistics was performed using Welch's unpaired *t* test.

### CPM was downregulated in CRC and inhibited CRC migration and invasion

**Figure 3:**
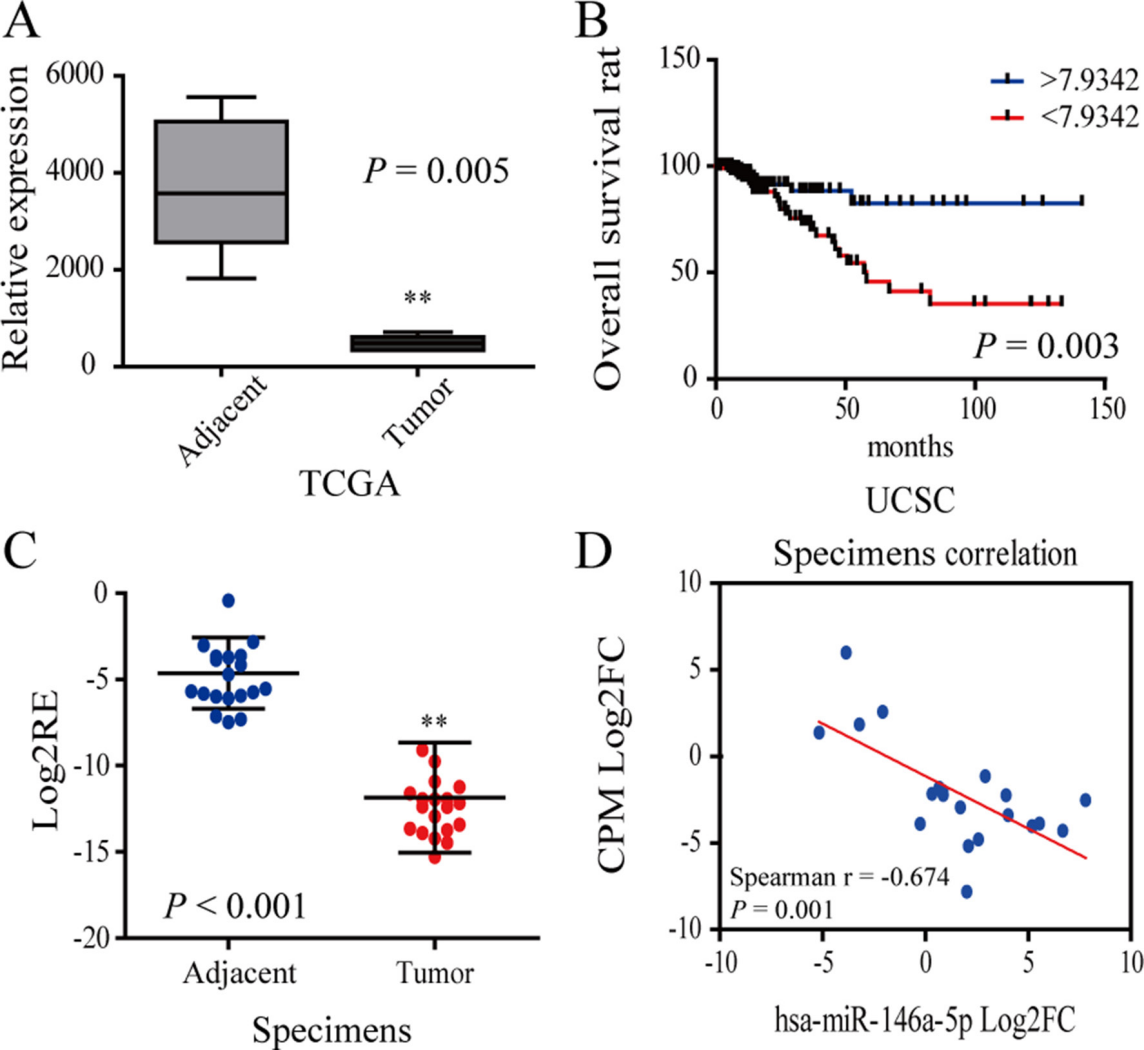
CPM was downregulated in CRC (**A**) The relative expression of CPM in the TCGA database. (**B**) Survival curve of CRC patient information from the UCSC database. The blue line represents increased CPM expression (Log2FC > 7.9342), whereas the red line represents reduced CPM expression (Log2FC < 7.9342). (**C**) Log2RE (relative expression) of CPM was detected in 20 pairs of specimens from CRC patients (RT-PCR). (**D**) Correlation of CPM expression and miR-146a-5p in 20 pairs of CRC specimens.

According to the data obtained from TCGA, Oncomine and UCSC database, we found that the expression of CPM was significantly reduced and decreased expression of CPM was associated with worse prognosis (Figure 3A, 3B). Meanwhile, we tested 20 pairs of CRC patient specimens using qRT-PCR and found that the CPM expression was significantly lower in tumor tissues than in the adjacent tissues in 95% of the specimens, in consistence to the results of bioinformatics analyses (Figure 3C). The expression of CPM and miR-146a-5p was negatively correlated in most paired CRC specimens (Figure 3D).

**Figure 4:**
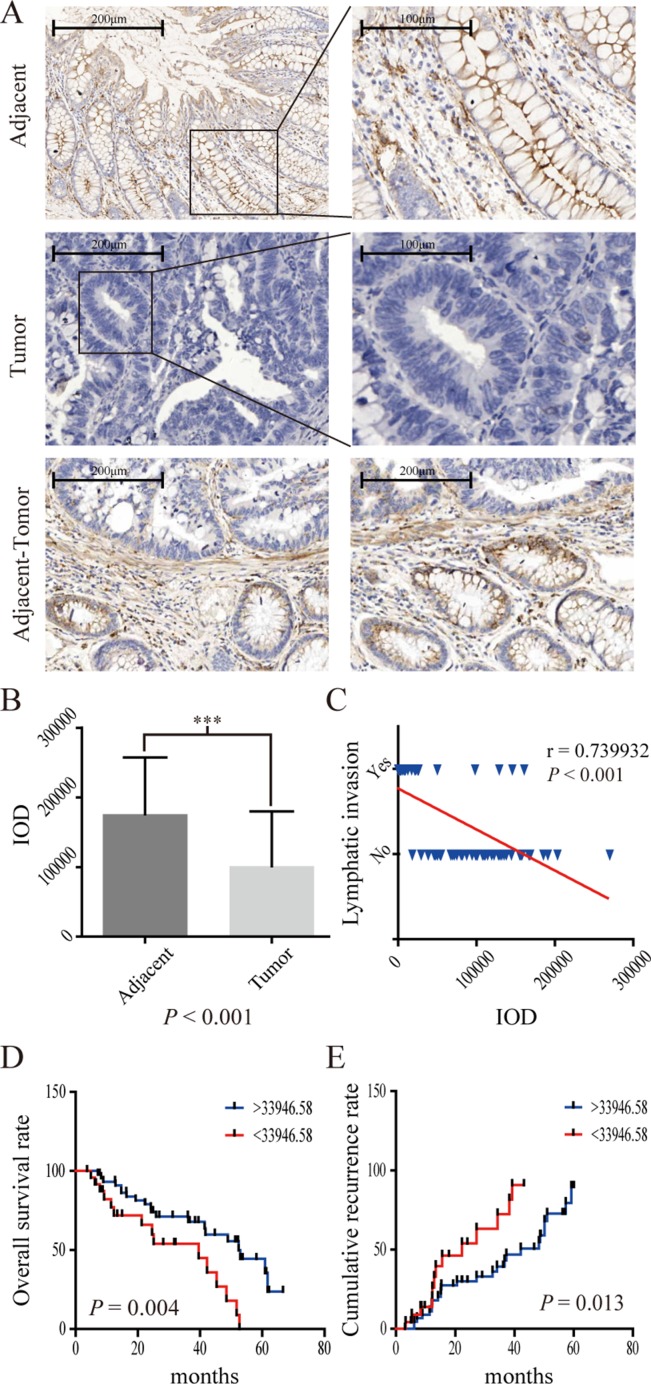
IHC results of CPM in 70 pairs of CRC specimens (**A**) CPM expression in adjacent specimens, CRC specimens and adjacent-tumor tissues; (**B**) IOD (integrated optical density) results of CPM expression in 70 pairs of specimens. (**C**) CPM expression was negatively correlated with lymph node invasion in 70 pairs of specimens. (**D**) Survival curve of 70 CRC patients. The blue line represents increased CPM expression (IOD > 33,946.58), whereas the red line represents reduced CPM expression (< 33,946.58). (**E**) Recurrence curve of 70 CRC patients. The blue line represents reduced CPM expression (IOD > 33,946.58), whereas the red line represents increased CPM expression (< 33,946.58).

The IHC results from 70 CRC patients also showed that CPM was obviously reduced in tumor tissues. In contrast, CPM accumulated at the cell membrane of normal colonic gland tissues (Figure 4A, 4B). Decreased expression of CPM was also associated with lymphatic invasion (*P* < 0.001)( (Figure 4C) and worse prognosis (*P* = 0.004, threshold = 33,946.58) (Figure 4D), while a relative increase in the expression of CPM was associated with a lower recurrence rate (Figure 4E). Moreover, the CPM expression level in the 70 CRC patient samples correlated with N stage (*P* < 0.001),, number of positive lymph nodes (*P* = 0.014), pathological grade (*P* = 0.033), tumor differentiation (*P* = 0.027) and AJCC stage (*P* < 0.001) but was not related to age, gender, pathologic type, anatomic neoplasm subdivision, venous invasion, T stage, M stage, serum CEA, CA19-9, CA125 and tumor size (Supplementary Table 1).

**Figure 5:**
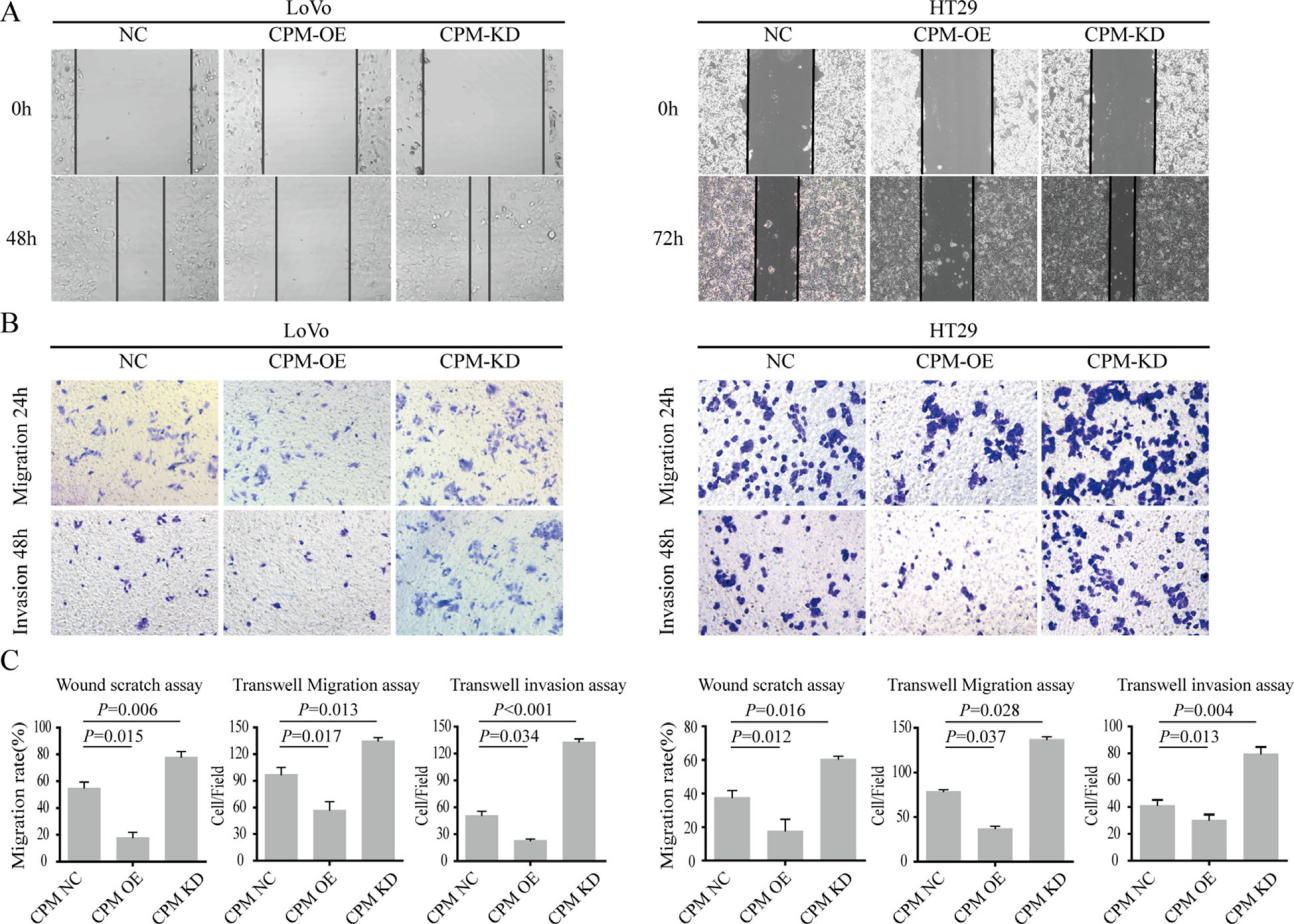
CPM inhibited CRC metastasis and invasion (**A**) Results of scratch wound cell migration assays for LoVo-CPM NC/OE/KD and HT29-CPM NC/OE/KD cells. (**B**) Results of transwell migration assays (upper layer) and transwell-matrigel invasion assays (lower) for LoVo-CPM NC/OE/KD and HT29-CPM NC/OE/KD cells. (**C**) The statistical results of scratch wound cell migration assays, transwell migration assays and transwell-matrigel invasion assays.

We next overexpressed CPM in LoVo and HT29 cells and found that the migration and invasion cells were significantly inhibited (*P* < 0.05). Then, we knocked down CPM in HT29 and LoVo cells and found that the migration and invasion abilities were upregulated (Figure 5).

### MiR-146a-5p modulated CPM-Src-FAK signaling

As previously reported, CPM was likely responsible for the initial metabolism of EGF, while FAK and c-Src (also known as Src) have been shown to form a transient, active complex following integrin engagement by EGF receptors [8]. Therefore, in this research, we hypothesized that the conversion of EGF by CPM affected its combination with EGFR and further affect the EGFR/Src/FAK signaling pathway in CRC. In this study, we focus on the impact of miR-146a and CPM on the SRC/FAK axis. Effect of CPM on the EGF/EGFR axis will serve as the focus of our future research.

**Figure 6:**
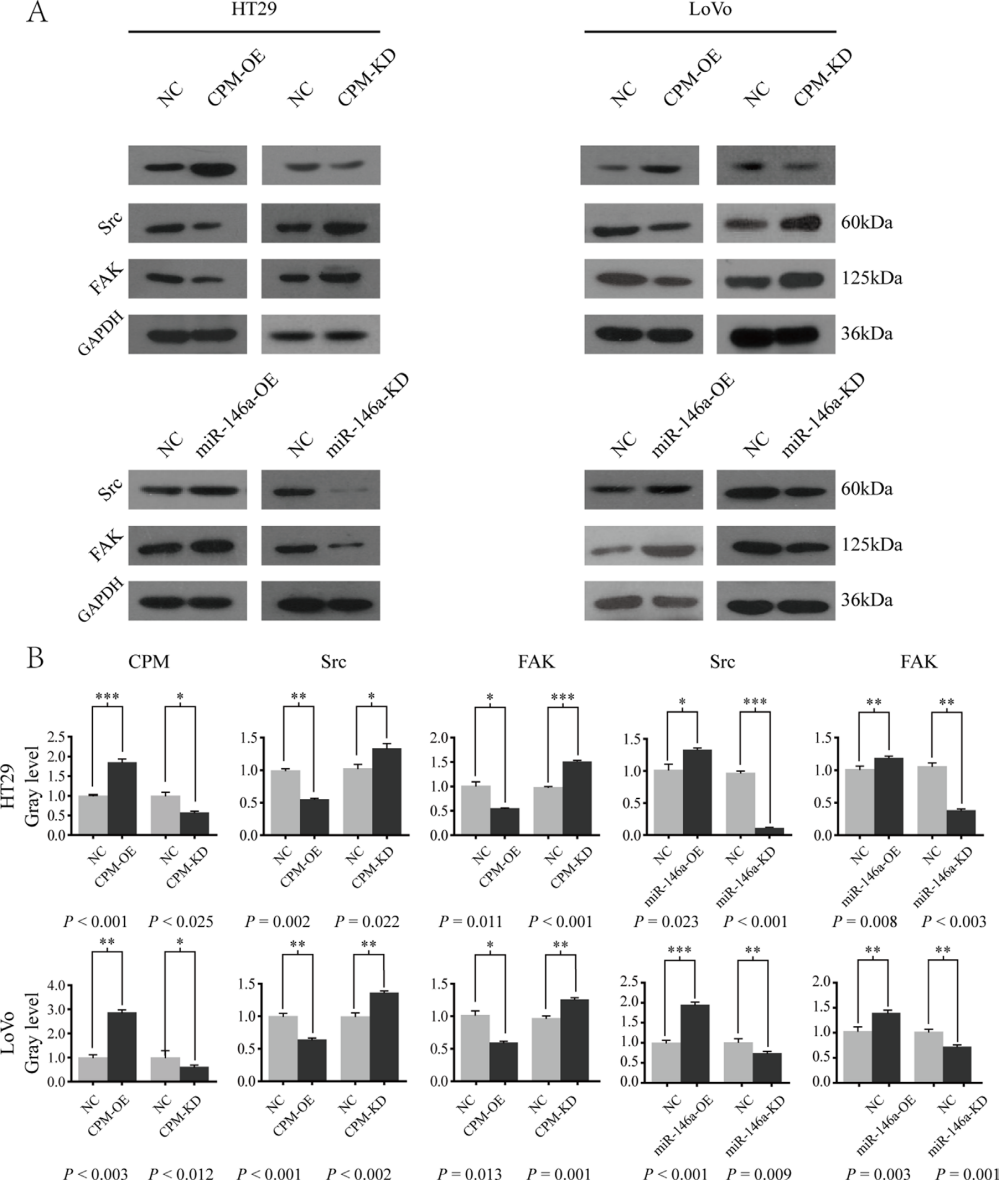
miR-146a-5p modulated CPM-Src-FAK signaling pathway (**A**) Western blot results of CPM, Src and FAK in HT29-CPM-NC/OE/KD and HT29-miR-146a-5p-NC/OE/KD cells. Src and FAK in LoVo-miR-146a-5p-NC/OE/KD and LoVo-miR-146a-5p-NC/OE/KD cells. (**B**) Gray level of the Western blot results for LoVo-CPM-NC/OE/KD, LoVo-miR-146a-NC/OE/KD, HT29-CPM-NC/OE/KD, and HT29-miR-146a-NC/OE/KD cells.

We overexpressed CPM in LoVo and HT29 cells and found that the expressions of Src and FAK were significantly inhibited (*P* < 0.050). Then, we knocked out CPM in HT29 and LoVo cells and found that Src and FAK expressions were enhanced compared with those in the controls (*P* < 0.050) (Figure 6). Furthermore, the expressions of Src and FAK (PTK2) were positively correlated with the N and M stage of CRC in the UCSC database (*P* < 0.050) (Supplement Figure 2A). In addition, the expression of CPM in the UCSC database exhibited a negative correlation with Src (Spearman correlation coefficient −0.296, *P* = 0.019), while the negative correlation between the expression of CPM and FAK was not significant (Spearman correlation coefficient −0.235, *P* = 0.066) (Supplementary Figure 2B). No significant correlation was noted between the expression of miR-146a and Src (Spearman correlation coefficient 0.497, *P* = 0.050)/FAK (PTK2) (Spearman correlation coefficient 0.129, *P* = 0.63) in the UCSC database.

## DISCUSSION

The results of our study showed that miR-146a-5p was upregulated and promoted cell migration, invasion in human CRC. Hwang WL, Jiang JK, Yang SH et al. reported that miR-146a knockdown in sphere-derived cancer stem cells (SDCSCs) reduced their sphere-forming ability, clonogenicity and anchorage-independent growth without affecting cellular proliferation and viability. Kuipers et al. reported that miR-146a repression attenuated tumorigenesis and metastasis *in vivo* [9], which was consistent with our findings.

We used bioinformatics and luciferase assays to demonstrate that CPM was the direct target gene of has-miR-146a. CPM is a membrane-bound, zinc-dependent protease that cleaves C-terminal basic residues, such as arginine or lysine, from peptides/proteins [10]. Anguraj Sadanandam showed that CPM is highly expressed in colorectal enterocytes [11]. Given its wide distribution in human tissues, CPM is believed to play important roles in the control of peptide hormone and growth factor activity at the cell surface and in the membrane-localized degradation of extracellular proteins [12]. In recent years, CPM has emerged as a potential cancer biomarker [16]. In this study, we found that CPM expression was lower in CRC tissues than that in adjacent tissues. CPM expression in CRC specimens was also positively correlated with overall survival and negatively correlated with recurrence, N stage and lymph node invasion.

CPM, both in its membrane-bound and soluble forms, was likely responsible for the initial metabolism of EGF and the appearance of des-Arg^53^-EGF in interstitial and biological fluids [13]. The significance of the conversion of EGF to des-Arg^53^-EGF in biological fluids or on target cells was not clear and it could either exhibit additional activities or be further degraded [14]. Activation of ErbB2 (This gene encodes a member of the epidermal growth factor receptor family) and downstream signaling pathways can lead to increased Src protein synthesis and decreased Src protein degradation resulting in Src up-regulation and activation, which play critical roles in ErbB2-mediated breast cancer invasion and metastasis [15]. More commonly found in colon cancer is elevated expression of wild-type (wt) c-Src (also known as Src). Indeed, elevated protein levels and/or catalytic activity of c-Src have been detected in a number of human cancers, including lung, skin, colon, breast, ovarian, endometrial, and head and neck malignancies [16]. c-Src also interacts with focal adhesion kinase (FAK) that is postulated to play a key role in cancer metastasis by modulating the formation and turnover of focal adhesions [17]. Exploitation of FAK activity in human tumors occurs through elevated expression, a situation that correlates with increased cancer cell motility, invasiveness, and proliferation [18]. FAK and c-Src have been shown to form a transient, active complex following integrin engagement by EGF receptors [8].

In our study, we found that inhibition of miR-146a-5p and the overexpression of CPM led to the downregulated Src and FAK expression, whereas the overexpression of miR-146a-5p and the inhibition of CPM upregulated Src and FAK. UCSC data indicate that the expression of Scr and FAK (PTK2) is positively correlated with N and M stage in CRC, whereas the expression of CPM is negatively correlated with Src. We hypothesized that miR-146a-5p-CPM regulates the Src-FAK pathway and affects CRC migration.

Src is frequently deregulated in human colorectal cancer (CRC), and Src increased activity has been associated with poor clinical outcomes [19, 20]. Normal Src overexpression can induce high invasiveness of advanced cancer cells *in vitro*, which is consistent with the proposed role for Src in CRC metastasis [21]. Hong Ji and David W reported that Src (v-Src) was expressed in exosomes in CRC. In particular, the study demonstrated higher v-Src levels in exosomes from a metastatic cell lines (SW620) versus non-metastatic cell lines (SW480) [22, 23]. Src-FAK is part of a complex network of intracellular signals that are important in regulating cancer cells [24]. Wu JC and Chen YC reported that the Src_SH2 domain was regulated by FAK, and the FAK-dependent association of the Src_SH2 domain was necessary and sufficient for Src FA targeting [25, 26], and inhibition of Src targeting into FAs significantly reduced cell migration [27]. FAK is a widely expressed cytoplasmic protein tyrosine kinase involved in integrin-mediated signal transduction. FAK plays an important role in the control of several biological processes, including cell spread, migration, and survival [28]. In CRC samples, FAK was overexpressed, and positive FAK expression correlated with lymph node metastasis and cellular differentiation [17]. Increased dosage of the FAK gene was also observed in many cell lines derived from human tumors of the lung, breast and colon [18]. In summary, our study showed that miR-146a-5p was upregulated in CRC and promoted migration and invasion *in vitro*. Moreover, CPM was identified as a direct target of miR-146a-5p. CPM, which was downregulated in CRC, also exhibited the potential to inhibit the migration and invasion, and affected the prognosis of CRC. Our results showed that CPM and miR-146a could regulate the expression of Src and FAK. Thus, we hypothesize that miR-146a promotes the migration and invasion of CRC by inhibiting CPM, thereby inducing Src and FAK upregulation.

## MATERIALS AND METHODS

### Patients and tissue specimens

Seventy pairs of samples from CRC patients were included in this study for immunohistochemistry (IHC), and an additional 20 pairs of CRC patient samples were included for qRT-PCR. All patients were pathologically diagnosed. Patients with a history of other solid tumors, radiotherapy and chemotherapy were excluded. Suitable formalin-fixed paraffin-embedded (FFPE) tissue samples were subjected to clinicopathological examination, and follow-up data were available for all patients. The tumor stage was defined in accordance with the AJCC staging system (2009 version).

### Bioinformatics analysis

The differences in miR-146a-5p, CPM, Src and FAK (PTK2) expression were analyzed using the TCGA and UCSC Cancer Browser (https://genome-cancer.ucsc.edu/proj/site/hgHeatmap) databases. Then, the target gene of miR-146a-5p was locked using a combination of Targetscan and at least two of the other five algorithms (miRanda, miRDB, miRWalk, RNA22, and SUM) compiled with at least three of the six algorithms (miRanda, miRDB, miRWalk), The Spearman correlation coefficient between the Log2FC of these candidate targets and the Log2FC of miR-146a-5p was calculated according to the data fromUCSC Cancer database. We then assessed whether these candidate target genes were downregulated in CRC tissues by referencing the Oncomine database. The target was downregulated more than 20 data sets for statistical significance of *P* < 0.05, FC ≥ 2. Candidate target genes were selected by RT-PCR to identify the final target genes (Supplementary Figure 1A).

### Cell line culture

Five CRC cell lines (HT29, SW480, LoVo, RKO, and HCT116) and an immobilized colonic epithelial cell line (FHC) were included in this study. Each of the cell lines was maintained in 90% RPMI 1640 (Invitrogen, Carlsbad, CA) supplemented with 10% fetal bovine serum.

### Lentiviral vectors and transfection

Lentiviral vectors for human miR146a-5p mimics/inhibitor carrying a green fluorescent protein (GFP) sequence, CPM knockdown, and CPM overexpression were constructed by Hanyin Co. (Shanghai, China). The recombinant lentivirus and the negative control (NC) lentivirus (GFP-lentivirus; Hanyin Co. Shanghai, China) were prepared and titered to 10^9^ TU/mL (transfection units). To obtain the stable cell line, cells were seeded in 6-well dishes at a density of 2 × 10^5^ cells per well. The cells were infected with the same viral titer with 8 μg/mL polybrene the following day. Approximately 72 h after viral infection, GFP expression was confirmed under a fluorescence microscope. The culture medium was replaced with selection medium containing 4 μg/mL puromycin. The cells were cultured for at least 14 days. The puromycin-resistant cell clones were isolated, amplified in medium containing 2 μg/mL puromycin for 7 to 9 days, and transferred to medium without puromycin. The clones were designated KD or OE cells.

### Real-time PCR

SYBR-green based quantitative real-time PCR was conducted to assay relative miRNA and mRNA levels using the 7500 PCR system. For mature hsa-miR-146a-5p and CPM expression analyses, gene-specific primers were purchased from GeneCopoeia, China. hsnRNA U6, and *GAPDH*.

### Protein preparation and Western blot

Cells were harvested and lysed in ice-cold RIPA (20 mmol/L Tris; pH 7.5). The protein concentration was quantified using the BCA protein assay from Pierce Bioscience (Vazyme E112-01/02 Nanjing China). The protein lysates (35 μg) were separated by SDS-PAGE and transferred to PVDF membranes (Hybond-P, Amersham). After blocking, the membranes were incubated with rabbit anti-CPM (Abcam, ab150405, UK), rabbit anti-Src (Abcam, ab109381), or rabbit anti-FAK antibody (Abcam, ab76496). Anti-GAPDH antibody (Vazyme, Ab103-02) was used as a loading control. After incubated with HRP-conjugated secondary antibodies, the blots were visualized using enhanced chemiluminescence (NCM Biotech P10200).

### Immunohistochemistry

Immunohistochemistry (IHC) was performed on tissues fixed with formaldehyde and embedded in paraffin wax. After deparaffinization and rehydration, the endogenous peroxidase activity was blocked and antigen retrieval was performed. The primary rabbit antibody (anti-CPM; 1/100 dilution; abcam ab150405) was incubated overnight at 4°C. After carefully washing and incubation with specified HRP-conjugated secondary antibody, protein expression was detected using 3,3N-diaminobenzidine tertrahydrochloride (DAB).

### Luciferase assay

A reporter vector containing the human CPM 3′UTR was cloned. Two putative miR146a-5p binding elements in the region (769–775 and 2458–2464) were mutated from AGTTCTC to GTCCAGA by site-directed mutagenesis. LoVo (2.5 × 10^5^ cells) cells were seeded onto a 24-well dish. The next day, cells were transfected with the reporter and effector constructs using the Fugene HD reagent according to the manufacturer's protocol. After 48 h, a luciferase assay was performed using the dual-luciferase reporter assay system (Promega).

### Scratch wound cell migration assay

The wound-healing assay was performed by seeding cells into 6-well plates at 2 × 10^5^ cells/well, and the cells were cultured for 16 to 24 h until reaching ~90% confluency. Then, a scratch wound was made, and the culture medium was changed to the appropriate medium without FBS. The wound closure was detected at 0, 24, 48 h and 72 h. Each experiment was repeated at least three times.

### Transwell migration assay/transwell-matrigel invasion assay

Cells were seeded at 2 × 10^5^ cells/well in the upper chamber of 8-μm pore-size Transwell 24-insert plates (Corning, NY, USA) with appropriate medium without FBS. The upper chambers were coated previously with 30 μg Matrigel (BD Biosciences, San Jose, CA, USA for Transwell-Matrigel invasion assay only), and the lower chamber contained appropriate medium with 10% FBS. After 48 h, cells on the bottom of the inserts were fixed in 4% paraformaldehyde and stained with 0.05% crystal violet. Then cells that invaded into the lower surface were counted in at least 10 fields using an 200× microscope (Olympus). Each experiment was repeated at least three times.

### Soft agar colony formation test

Cells were harvested and pipetted to single-cell suspension in complete culture media to a volume of 1 × 10^6^/mL. 1640 medium containing 10% FBS and 0.75% agar was added to 6-well plates at 3 mL per well until solidification at room temperature. Then 3 × 10^4^ cells in 3 mL of 1640 medium containing 10%FBS and 0.36% agar were added to the top layer. The dish was incubated at 37°C for 3 weeks, followed by staining with 0.1% crystal violet.

### Statistical analysis

The data were analyzed using SPSS 13.0 software and graphpad (http://www.graphpad.com/quickcalcs/contMenu/). Welch's unpaired *t-test* and survival analysis were used. A *P value* < 0.05 represented a significant difference.

## SUPPLEMENTARY MATERIALS FIGURES AND TABLES


