# Supplementary material for: MicroRNA-146a promote cell migration and invasion in human colorectal cancer via carboxypeptidase M/src-FAK pathway
Source: Oncotarget. 2017 Feb 7;8(14):22674–84. doi: 10.18632/oncotarget.15158 (PMC5410254; doi:10.18632/oncotarget.15158)
Supplement: Supplementary file 1 [file oncotarget-08-22674-s001.pdf]

MicroRNA-146a promote cell migration and invasion in human colorectal cancer via carboxypeptidase M/src-FAK pathway

Supplementary Materials

qRT-PCR results for candidate target genes

qRT-PCR results in cell lines showed that CPM regulated by miR-146a-5p was the most significant in these four genes after the expression of miR-146a-5p was altered.

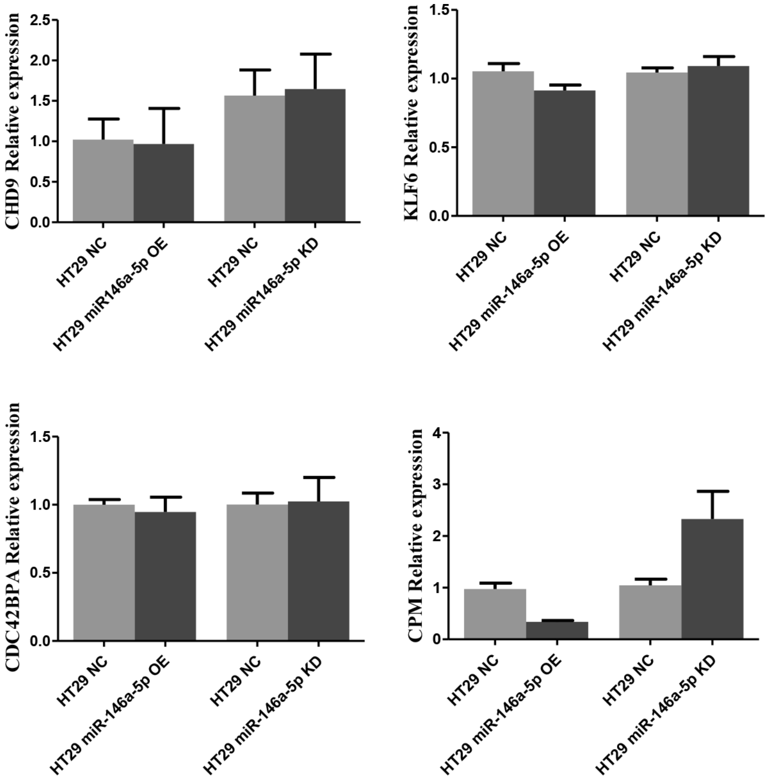

$P = 0.011$ $P = 0.019$

(We detected rt-PCR in FHC, HT29, and LoVo cell lines after transfection with miR-146a-5p NC, OE, and KD, the result were similar in three groups, here we present the results of HT29- miR-146a-5p NC, OE, and KD, the remaining two groups did not described here).

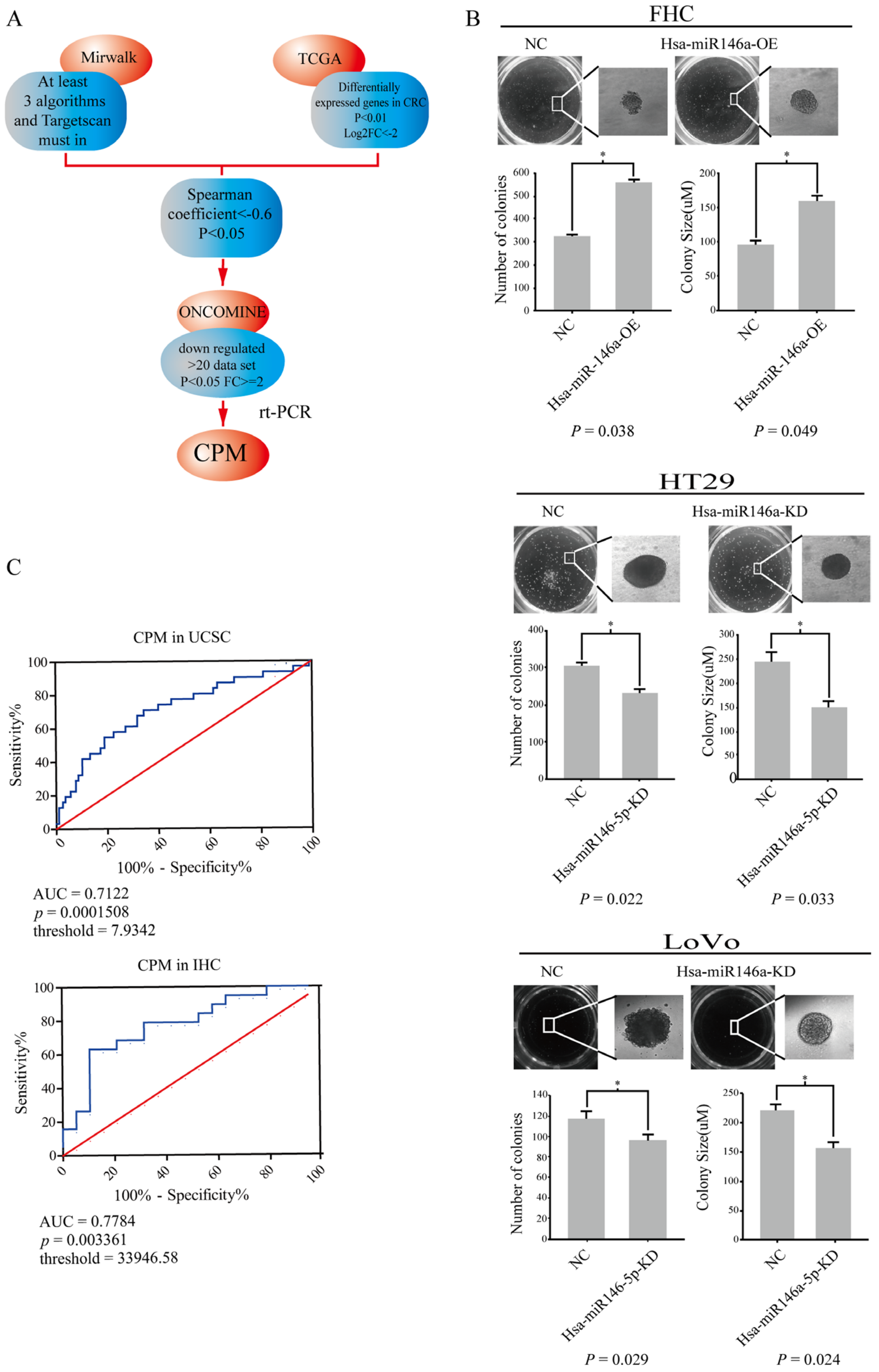

Supplementary Figure 1: (**A**) Method for searching targets of miRNAs. (**B**) Colony formation of FHC miR-146a-5p NC/OE, HT29 miR-146a-5p NC/KD and LoVo miR-146a-5p NC/KD cells. (**C**) The ROC curve of CPM expression in the UCSC database and the ROC curve for the IOD of CPM expression in 70 CRC specimens.

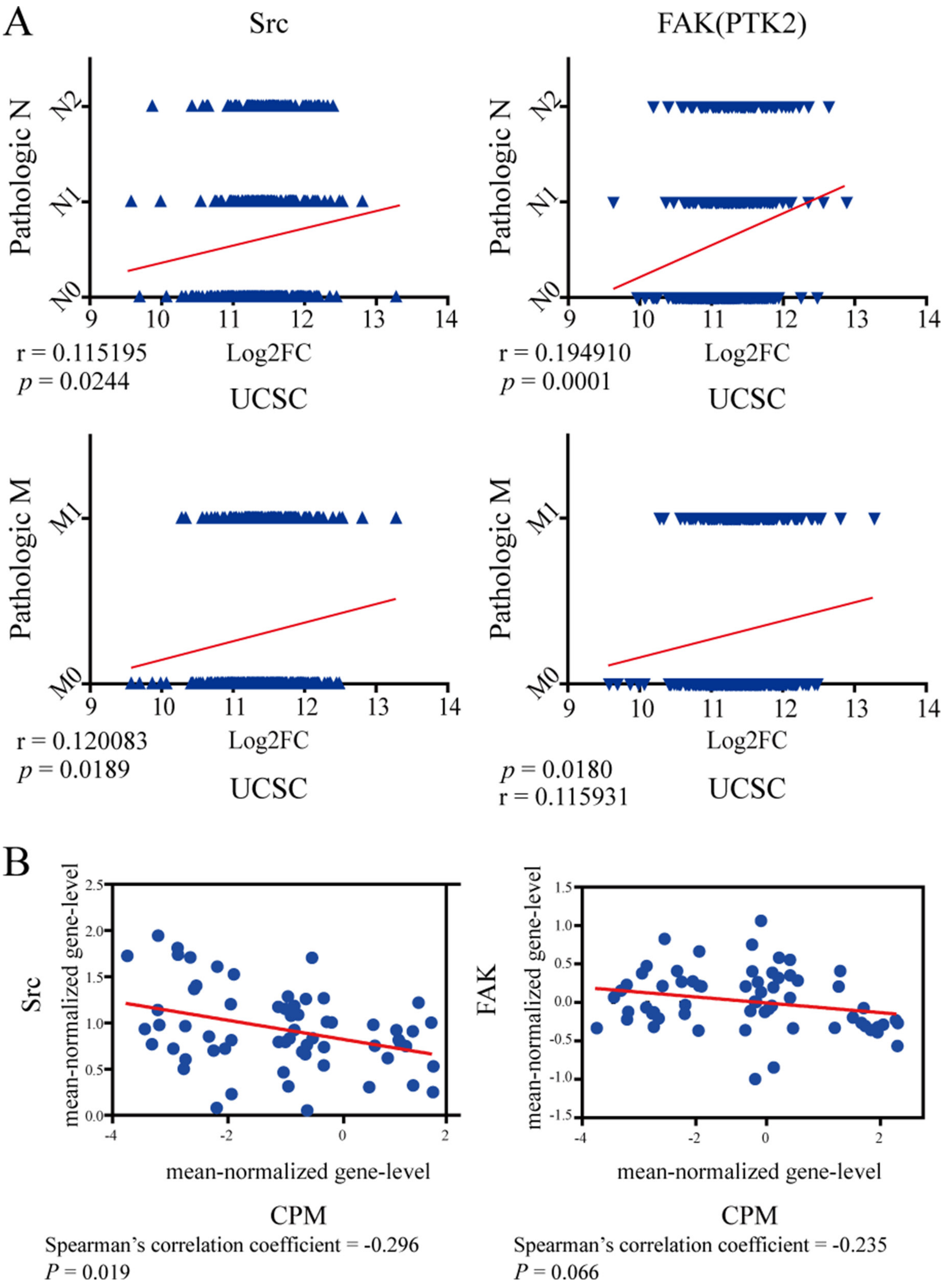

Supplementary Figure 2: (**A**) Src and FAK expression was positively correlated with N/M stage of CRC in the UCSC database. (**B**) Correlation between the expression of CPM and Src and that between CPM and FAK.

Supplementary Table 1: Correlation between CPM expression and cliniopathological features in 70 CRC

Variables	CPM IOD		*P* value
	High expression	Low expression	
Age (years)			
≥ 55	32	18	1
< 55	13	7	
Gender			
Male	23	15	0.642
Female	22	10	
Pathologic type			
Adenocarcinoma	43	20	0.096
Mucinous adenocarcinoma	2	5	
Pathological grade			
I/II	41	17	0.033
III/IV	4	8	
Pathologic N			
N0	40	6	0.000
N1/2	5	19	
Pathologic M			
M0	45	22	0.079
M1	0	3	
Pathologic T			
T1/2	11	3	0.350
T3/4	34	22	
AJCC stage			
I/II	41	7	0.000
III/IV	4	18	
Tumor differentiation			
I	3	7	0.027
II	35	17	
III	7	1	
Lymphatic invasion			
Yes	5	19	0.000
no	40	6	
Number of lymphnodes positive			
≥ 5	2	7	0.014
< 5	43	18	
Anatomic neoplasm subdivision			
Left colon	10	9	0.47
Right colon	11	6	
Transverse Colon	2	0	
Rectum	22	10	
Venous invasion			
Yes	3	3	0.750
no	42	22	
Serum CEA (U/ml)			
≥ 5	19	9	0799
< 5	26	16	
Serum CA19-9 (ng/ml)			
≥ 37	9	7	0.641
< 37	36	18	
Serum CA125 (U/ml)			
≥ 35	7	4	1
< 35	38	21	
Tumor size (diameter, cm)			
≥ 4	30	21	0.200
< 4	15	4	
